# Determinants of Perceived Own Risk for Developing Dementia and the Perception That Memory Deterioration Is Preventable. Findings From the General Adult Population in Germany

**DOI:** 10.3389/fmed.2020.00203

**Published:** 2020-06-09

**Authors:** André Hajek, Hans-Helmut König

**Affiliations:** Department of Health Economics and Health Services Research, University Medical Center Hamburg-Eppendorf, Hamburg, Germany

**Keywords:** risk perception for developing dementia, perceived modifiability of memory deterioration, dementia prevention, GSOEP, fear of dementia, dementia worry, chronic diseases

## Abstract

**Objective:** To identify among the general population the determinants of (1) perceived own risk of developing dementia and (2) the perception that memory deterioration is preventable.

**Methods:** For this study, cross-sectional data were taken from the German Socio-Economic Panel (innovation sample, year 2012), which is a population-based, longitudinal study of German households. There were 1,542 individuals included in our analytical sample.

**Results:** Multiple linear regressions showed that an increased perceived own risk of developing dementia was associated with younger age, higher education, poor self-rated health, an increased number of chronic diseases, and an increased agreement that a diagnosis of dementia would ruin their life. An increased perceived modifiability of memory deterioration was associated with higher education, and not being employed, but not health-related variables.

**Conclusion:** Several determinants of the perceived own risk of developing dementia and the perceived modifiability of memory deterioration have been identified. Addressing modifiable factors may be beneficial for changing these outcome measures.

## Introduction

At present, ~1.7 million individuals in Germany live with dementia (1). According to recent calculations, it is expected that this number will increase to roughly 3 million individuals in 30 years (1). Approximately 4 of 10 individuals 90 years or older suffer from dementia (1), whereas fewer than 4 of 100 individuals aged 70 to 74 years have dementia.

Often caused by Alzheimer disease (AD), dementia is a syndrome characterized by progressive deterioration of cognitive function (particularly memory function). During the course of the disease, performing daily activities becomes more and more difficult. Hence, individuals with dementia often need a considerable amount of care (2).

Despite the fact that previous risk factors for dementia have been identified, such as lifestyle or genetic factors, not everyone is familiar with the causes of dementia and how to prevent dementia (3). For example, based on data from the German general population, ~55% of the individuals think that dementia can be prevented, leaving 45% believing that dementia is not preventable (4). Given demographic aging, a very high prevalence rate of dementia among very old individuals, and the only moderately strong belief that memory deterioration is preventable, it is plausible that individuals may think that they have a high risk of developing dementia.

The perception among individuals that they have a high risk of developing dementia may result in a markedly decreased subjective well-being (5). This is because these individuals may fear the serious, life-changing consequences of dementia. Furthermore, it may result in feelings of despair or hopelessness (6). Furthermore, we assume that they may be afraid of future stigmatization or social isolation (7). Therefore, knowledge about the determinants of perceived own risk of developing dementia is important.

A weak belief that memory deterioration is preventable may ultimately result in an unhealthy lifestyle, as these individuals may perceive efforts to live a healthy life as pointless. Individuals with a weak belief that memory deterioration is preventable may also have a high external locus of control. This means that these individuals tend to believe that their health is based on external factors (e.g., fate or luck) (6). Ultimately, such individuals may have a higher probability of developing dementia as part of a self-fulfilling prophecy. That is, the belief that memory deterioration is not preventable becomes true because individuals behave in accordance with this belief (i.e., they invest little in preventing memory deterioration, i.e., by adapting their lifestyle).

To date, only one recent study has examined dementia risk and protective factor awareness among older adults (8). In a study conducted in New Zealand, 47% of individuals felt that they were at a future risk of developing dementia. Moreover, 95% of the analyzed individuals felt that this would change their lives in a significant way. Furthermore, 91% of individuals thought that lifestyle changes could decrease their risk of dementia, and 88% of individuals thought that they could make the changes required. Another study (online survey, with n = 590 individuals residing in private households aged 40–75 years; Province of Limburg, the Netherlands) showed that 56% of the individuals were unaware of a link between lifestyle factors and dementia risk (9).

Moreover, very few studies have examined the perceived risk of developing dementia and beliefs about the effectiveness of options for reducing risk of and treating AD [taking place in the United States and Australia (6, 10, 11)]. However, only one study is based on a nationally representative sample of older adults (11). Furthermore, there is a lack of studies investigating the determinants of perceived own risk of developing dementia and the perception that memory deterioration is preventable in the general population in Germany. Therefore, our aim was to identify in the general population the determinants of (1) perceived own risk of developing dementia and (2) the perception that memory deterioration is preventable.

## Materials and Methods

### Sample

Data were drawn from the German Socio-Economic Panel (GSOEP), located at the German Institute for Economic Research, DIW Berlin (with the first wave taking place in the year 1984). The GSOEP is a well-known nationally representative study of adult (≥17 years) inhabitants in Germany (with ≥11,000 households and >20,000 individuals), who participate every year in the interviews. Various topics are included in the GSOEP, such as political preferences, occupational history, and well-being. Low survey attrition (12) and very high response rates (13) have been demonstrated for the GSOEP [for further details regarding the GSOEP, see (14)]. Dementia-related questions (our outcome measures) were measured only in the GSOEP innovation sample (GSOEP-IS) in 2012. Like the GSOEP, the GSOEP-IS is a representative sample of the community-dwelling adult population in Germany (15). Beginning in 2011, the GSOEP-IS takes place every year, covering some core questions. In addition, the GSOEP-IS includes measures solely developed by the users of the GSOEP (i.e., the scientific community). Based on a competitive referral process, high-quality tools are selected on the basis of their relevance to the sample.

Because we focused on dementia-related outcome measures (described in further detail in the following section), cross-sectional data from the GSOEP-IS of 2012 were used. Hence, our analytical sample totaled *n* = 1,542.

Informed consent was provided by all participants. An ethical approval was not obtained as requirements for the need of an ethical statement were not met (such as the risk for the respondents, examination of patients, lack of information about the aims of the study). Nevertheless, the German Council of Science and Humanities (“Wissenschaftsrat”) evaluated the GSOEP survey and approved it.

### Dependent Variables

Outcome measures were as follows:

1. How do you estimate your risk of getting a form of dementia, such as Alzheimer disease 1 day (1 = very low, 2 = low, 3 = average, 4 = increased or 5 = high)?
2. There is quite a lot of what I can do by myself to keep my mind from reduction (agreement with the statement: from 1 = *does not apply at all* to 7 = *fully applies*).

Similar measures have been used in other recently published articles (8, 11).

### Independent Variables

Various socioeconomic variables were included in our regression model: gender, age, family status (married, living separated from spouse; widowed; single; divorced; married, living together with spouse), labor force participation [not employed; employed (including full-time employment; regular part-time employment; vocational training; marginal employment; near retirement, zero working hours; community service; sheltered workshop; military service)], and educational level (International Standard Classification of Education (ISCED-97) (16); ranging from low education (ISCED 0–2), medium education (ISCED 3–4) and high education (ISCED 5–6)] in the regression model. Moreover, the number of chronic illnesses [count score: diabetes; asthma; cardiac disease (also: cardiac insufficiency, weak heart); cancer; stroke; migraine; high blood pressure; depressive disorder; joint diseases (including arthritis/rheumatism); chronic backache; sleep disorder; other illness)] and self-rated health (from 1 = very good to 5 = very bad) were included. In addition, the following (dementia-related) statement was included in our regression model as an independent variable: The diagnosis of dementia such as Alzheimer would ruin my life (from 1 = *does not apply at all* to 7 = *fully applies*).

### Statistical Analysis

Sample characteristics were calculated for our analytical sample. Following this, multiple linear regression models were used to investigate the determinants of perceived own risk of developing dementia and the perception that memory deterioration is preventable. Ordered probit regressions replaced linear regressions in sensitivity analysis. The level of significance was determined at *p* < .05. Statistical analysis was performed using Stata 16.0 (Stata Corp., College Station, TX, USA).

## Results

### Sample Characteristics

Descriptive statistics for our analytical sample are reported in Table 1. The mean age was 51.1 (*SD*, 17.2) years, and 47.6% of the individuals were male. Of the sample, 60.3% had a medium educational level. Further details are displayed in Table 1.

**Table 1:** Sample characteristics for analytical sample (GSOEP-IS, 2012; *n* = 1,542).

**Variables**	**Mean (*SD*)/*N* (%)**
Age: Mean *(SD)*	51.1 (17.2)
**Gender:** ***N*** **(%)**	
Male	734 (47.6%)
Female	808 (52.4%)
**Education:** ***N*** **(%)**	
Low education	244 (15.8%)
Medium education	929 (60.3%)
High education	369 (23.9%)
**Marital status:** ***N*** **(%)**	
Married, living together with spouse	934 (60.6%)
Other (married, not living together with spouse; divorced; widowed; single)	608 (39.4%)
**Employment status:** ***N*** **(%)**	
Employed (full-time employed; regular part-time employed; vocational training; marginally employed; near retirement, zero working hours; military service; community service; sheltered workshop)	880 (57.1%)
Not employed	662 (42.9%)
Self-rated health (from 1=“very good” to 5=“very bad”): Mean (*SD*)	2.5 (1.0)
Sum of chronic diseases: Mean (*SD*)	1.0 (1.2)
Agreement that a diagnosis of dementia would ruin his/her life (from 1=does not apply to 7 = fully applies): Mean (*SD*)	5.2 (1.9)
Perceived own risk for developing dementia (from 1 = very low to 5 = high): Mean (*SD*)	2.4 (0.9)
Perception that memory degradation is preventable (from 1 = does not apply to 7 = fully applies): Mean (*SD*)	5.8 (1.5)

### Main Regression Analysis

Results of multiple linear regressions are displayed in Table 2. *R*^2^ equaled 0.043 (with perceived own risk of developing dementia as the outcome measure) and 0.013 (with perceived modifiability of memory deterioration as the outcome measure). Because variance inflation factors were quite low in both regressions (with perceived own risk of developing dementia as the outcome measure: highest VIF was 2.08; with perceived modifiability of memory deterioration as the outcome measure: highest VIF was 2.09), multicollinearity was not a concern in our study.

**Table 2:** Determinants of (1) perceived own risk for developing dementia and (2) perception that memory degradation is preventable.

	**(1)**	**(2)**
**Independent variables**	**Perceived own risk for developing dementia**	**Perception that memory degradation is preventable**
Age	−0.01^***^	0.00
	(0.00)	(0.00)
Gender: Female (Ref.: Male)	0.06	0.09
	(0.05)	(0.08)
Marital status: Married, living together with spouse (Ref. Other)	0.06	−0.00
	(0.05)	(0.09)
Employment status: Not employed (Ref. Employed)	−0.04	0.20^*^
	(0.06)	(0.09)
Educational level:- Medium education (Ref. low education)	0.08	0.25^*^
	(0.07)	(0.11)
-High education	0.17^*^	0.25+
	(0.08)	(0.13)
Self-rated health (from 1 = “very good” to 5 = “very bad”)	0.09^**^	−0.08+
	(0.03)	(0.04)
Count score of chronic diseases	0.09^***^	0.06
	(0.03)	(0.04)
Agreement that a diagnosis of dementia would ruin his/her life (from 1 = does not apply to 7 = fully applies)	0.05^***^	0.01
	(0.01)	(0.02)
Constant	2.06^***^	5.36^***^
	(0.12)	(0.20)
Observations	1,509	1,542
*R*^2^	0.043	0.013

Results of multiple linear regression analysis. Unstandardized beta-coefficients are reported; robust standard errors in parentheses;

*** p < 0.001,

** p < 0.01,

* *p < 0.05, + p < 0.10; Perceived own risk for developing dementia ranges from 1 = very low to 5 = high; perception that memory degradation is preventable ranges from 1 = does not apply to 7 = fully applies*.

Multiple linear regressions revealed that an increased perceived own risk of developing dementia was associated with younger age (β = −0.01, *p* < 0.001), higher education (high education: β = 0.17, *p* < 0.05), poor self-rated health (β =.09, *p* < 0.01), an increased number of chronic diseases (β = 0.09, *p* < 0.001), and an increased agreement with the statement that a diagnosis of dementia would ruin their life (β = 0.05, *p* < 0.001). An increased perceived modifiability of memory deterioration was associated with higher education (medium education: β = 0.25, *p* < 0.01) and not being employed (β = 0.20, *p* < 0.05). It was not associated with health-related (including dementia-related) variables.

In a robustness analysis, multiple linear regressions were replaced by ordered probit regressions (results not shown, but available upon request). In terms of significance, findings remained very similar. However, it should be noted that the association between perceived modifiability of memory deterioration and employment status disappeared (*p* = 0.054), whereas the association between perceived modifiability of memory deterioration and high education became statistically significant (*p* < 0.05).

## Discussion

### Main Findings

The objective of our study was to identify in the general adult population the determinants of (1) perceived own risk of developing dementia and (2) the perception that memory deterioration is preventable. Multiple linear regressions revealed that an increased perceived own risk of developing dementia was associated with younger age, higher education, poor self-rated health, an increased number of chronic diseases, and an increased agreement with the statement that a diagnosis of dementia would ruin their life. An increased perceived modifiability of memory deterioration was associated with higher education and not being employed, but was not associated with health-related variables.

### Previous Research and Possible Explanations

Using nationally representative data from the adult population in Germany, this current study extends our current knowledge on the correlates of perceived own risk of developing dementia and the perception that memory deterioration is preventable. One particular benefit is that we demonstrated an association between an increased agreement with the statement that a diagnosis of dementia would ruin their life and an increased perceived own risk of developing dementia.

With regard to the perceived own risk of developing dementia (the first outcome measure of our study), another recent study has investigated the perceived lifetime risk of developing dementia based on nationally representative data from US adults aged 50 to 64 years (*n* = 1,019) (11). In this study, individuals were asked “How likely are you to develop dementia during your lifetime?” (very likely, somewhat likely, not likely). Among older adults, 48.5% reported that they were at least “somewhat likely” to develop dementia (“somewhat likely”: 44.3%, “very likely”: 4.2%). Regression analysis showed that, among other things, individuals with poor mental health reported a higher likelihood of developing dementia, whereas the perceived likelihood of developing dementia was not associated with fair or poor physical health. However, our findings are difficult to compare with the study conducted by Maust et al. (11) because our current study is based on data from individuals 17 years or older, whereas the study performed by Maust et al. (11) focused exclusively on individuals 50 to 64 years old in the United States.

In our study, an increased perceived own risk of developing dementia was associated with younger age, higher education, poor self-rated health, an increased number of chronic diseases, and an increased agreement with the statement that a diagnosis of dementia would ruin their life. Given the fact that various chronic illnesses are associated with subsequent cognitive decline (17, 18), the link between health-related variables (self-rated health and chronic diseases) and an increased perceived own risk of developing dementia appears to be very plausible.

It should be stressed that risk perceptions mainly refer to a cognitive judgment (19). Thus, individuals with higher education may more rationally evaluate their own risk of developing dementia (20). Furthermore, we assume that risk denial may occur more often in individuals with low education. Therefore, the association between higher education and an increased own risk of developing dementia is plausible to us.

It is well-known that the likelihood for developing dementia increases with age. Therefore, it is quite unexpected that an increased perception of own risk of developing dementia was associated with younger age in our study. However, this may be explained by (i) risk denial or (ii) by an increased dementia literacy among younger individuals (due to a higher education in younger individuals). Furthermore, while younger individuals may perceive the cognitive decline of older individuals, the older individuals themselves may not fully perceive this decline.

Lastly, the link between an increased agreement that a diagnosis of dementia would ruin their life and an increased perceived own risk of developing dementia may be explained by the life-changing nature of such an illness. That is, individuals may systematically overestimate the risk of developing dementia, because of its serious consequences. However, we have to acknowledge that this is a speculative assumption, and future research is required to test it.

With regard to the perception that memory deterioration is preventable (second outcome measure of our current study), only one recent study of individuals 50 years or older found that individuals show optimism about modifying dementia risk through lifestyle interventions (*n* = 216) (8). We are not aware of other studies investigating this outcome. Therefore, it is difficult to compare our results with other studies.

An increased perceived modifiability of memory deterioration was associated with higher education and not being employed. However, it was not associated with health-related variables. A possible explanation for the non-significant link between health-related variables and the perceived modifiability of memory deterioration may be provided by findings of a qualitative study. This study (focus group study conducted in 2011; 34 individuals aged between 52 and 90 years) (6) showed that responses about their perceived likelihood of developing dementia could be classified into (i) fear (majority of participants; individuals who avoid the question by expressing their fear instead), (ii) rational (mainly men; rationally estimated the likelihood of dementia by comparing their characteristics with the risk factors), and (iii) cynical perceptions (those who did not believe in risk factors). It may be the case that the non-significant link between health-related variables and the perceived modifiability of memory deterioration may be driven by a large proportion of individuals with decreased self-rated health and increased chronic conditions in our sample, who may have cynical perceptions of health as classified by Kim et al. (6).

The link between higher education and an increased perceived modifiability of memory deterioration appears to be plausible. Again, we assume that higher education is associated with increased rationality; in other words, individuals with high education may have a greater awareness of the risk factors of memory deterioration (20).

We think that the (counterintuitive) link between employment status and perceived modifiability of memory deterioration should be interpreted conservatively and with caution because this link did not achieve significance in sensitivity analysis (using ordered probit regressions). Future research is required to investigate this particular link in more detail. For example, this association could be examined in an analysis stratified by occupational class.

### Strengths and Limitations

As one of few studies on this topic, we examined the determinants of perceived own risk of developing dementia and the perception that memory deterioration is preventable in the general adult population. In our regression model, socioeconomic, health-related (including dementia-related) variables were included. However, as far as data are available, factors such as apolipoprotein E genotype or a family history of dementia should be included in future similar studies. Moreover, qualitative studies may deepen our knowledge about the factors associated with perceived risk of developing dementia. For example, the perceived risk may be a realistic assessment of one's lifestyle in a certain group of individuals, as suggested by Kim et al. (6). Other individuals who score high on cynical perceptions may deny possible risk factors (6). Although non-modifiable factors (such as age) are also important to investigate, future studies should focus on modifiable factors. Given the fact that it has been demonstrated that lifestyle changes could prevent or delay a third of dementia cases (21), investigating these modifiable risk factors is particularly important. Data were taken from a nationally representative sample (GSOEP-IS), which had very high response rates (13). Our outcome measures had a high face validity. Furthermore, similar measures were also used in other recent studies (8, 11). Nevertheless, our findings are based on cross-sectional data and should be validated by using longitudinal data, and based on more elaborated scales (for example, see the questionnaire used in the study conducted by Heger et al. (9). Furthermore, it might be of interest for future studies to examine whether our findings differ by cultural background.

### Conclusion and Future Research

Several determinants of the perceived own risk of developing dementia and the perceived modifiability of memory deterioration have been identified. Addressing modifiable factors may be beneficial for changing these outcome measures.

## Data Availability Statement

Publicly available datasets were analyzed in this study. GSOEP data access must comply with high security standards for maintaining confidentiality and protecting personal privacy. The data are also subject to regulations limiting their use to scientific purposes, that is, they are only made available to the scientific community (in German language only). GSOEP Data from the DIW Berlin are available on request, after completing a free contract with DIW, either via personalized encrypted download or via certified mail on a DVD. Interested researchers may contact the DIW Berlin via soepmail@diw.de. Please see for further Information: https://www.diw.de/en/diw_02.c.238237.en/conditions.html.

## Ethics Statement

Ethical review and approval was not required for the study on human participants in accordance with the local legislation and institutional requirements. The patients/participants provided their informed consent to participate in this study.

## Author Contributions

AH and H-HK: design and concept of analyses, preparation of data, statistical analysis and interpretation of data, preparing of the manuscript. Both authors critically reviewed the manuscript, provided significant editing of the article and approved the final manuscript.

## Conflict of Interest

The authors declare that the research was conducted in the absence of any commercial or financial relationships that could be construed as a potential conflict of interest.
